# The antiquity of Nullarbor speleothems and implications for karst palaeoclimate archives

**DOI:** 10.1038/s41598-018-37097-2

**Published:** 2019-01-24

**Authors:** Jon D. Woodhead, J. M. Kale Sniderman, John Hellstrom, Russell N. Drysdale, Roland Maas, Nicholas White, Susan White, Paul Devine

**Affiliations:** 1 School of Earth Sciences, University of Melbourne, Parkville, VIC 3010 Australia; 2 School of Geography, University of Melbourne, Parkville, VIC 3010 Australia; 3 Environnements, Dynamiques et Territoires de la Montagne, UMR CNRS, Université de Savoie-Mont Blanc, 73376 Le Bourget du Lac, France; 4 Victorian Speleological Association, GPO Box 5425, Melbourne, VIC 3001 Australia; 5 School of Life Sciences, La Trobe University, Bundoora, VIC 3086 Australia; 6 Speleological Research Group of Western Australia, P.O. Box 1611, East Victoria Park, WA 6981 Australia

## Abstract

Speleothems represent important archives of terrestrial climate variation that host a variety of proxy signals and are also highly amenable to radiometric age determination. Although speleothems have been forming on Earth for at least 400 million years, most studies rely upon the U-Th chronometer which extends only to the mid Pleistocene, leaving important questions over their longer-term preservation potential. To date, older records, exploiting the advantages of the U-Pb chronometer, remain fragmentary ‘snapshots in time’. Here we demonstrate the viability of speleothems as deep time climate archives by showing that a vast system of shallow caves beneath the arid Nullarbor plain of southern Australia, the world’s largest exposed karst terrain, formed largely within the Pliocene epoch, with a median age of 4.2 Ma, and that, in these caves, even the most delicate formations date from this time. The long-term preservation of regional-scale cave networks such as this demonstrates that abundant speleothem archives do survive to permit the reconstruction of climates and environments for much older parts of Earth history than the ~600 ka period to which most previous studies have been limited.

## Introduction

Speleothems (secondary cave calcite deposits including stalagmites, stalactites and flowstones) are increasingly recognised as critical sources of climate history. Their incremental growth provides a record of changing conditions above the cave in which they form, and they can be readily dated to high precision using the decay of natural U incorporated at formation. Since the first application of radiometric dating to speleothems^1,2^, they have become the source of some of our most detailed and precise reconstructions of past changes in Earth’s climate, ranging from glacial-interglacial to annual temporal scales^3–5^. However, the ~600 ka limit of the U-Th decay chain, the most widely employed chronometer, provides a biased view of the utility of speleothem archives and the cave levels/systems in which they form - a situation analogous to the radiocarbon limit, which has similarly restricted our understanding of Quaternary climate and environmental change. Individual speleothem records usually represent only relatively brief growth episodes, with few specimens encompassing more than 100 ka. This, combined with an apparent loss of older speleothems over time^6^, has effectively diverted attention away from an awareness that contemporary cave systems containing abundant speleothems have surely supported their growth in the past, long before the age limit of the dominant dating technique.

Because of this situation, the likely antiquity and magnitude of the unexplored speleothem archive, and the cave systems that support them, in general, remain highly uncertain. The U-Pb chronometer, which is able to date suitable minerals of any age, has been applied to a handful of speleothems demonstrating that, under certain conditions, ancient samples can be preserved from the Pleistocene^7–9^, Pliocene^10,11^, Early -and Mid-Miocene^12^ and even the Permian^13^ (Supplementary Fig. S1). At present, however, the small number of samples studied, their scattered distributions, and the fact that some of these are associated with palaeokarst and unroofed cave floors in highly eroded landscapes^12^, all give an impression that such ancient speleothems persist only sporadically/fortuitously or that they are likely to provide only fragmentary records of past landscapes, climates and environments.

**Figure 1:**
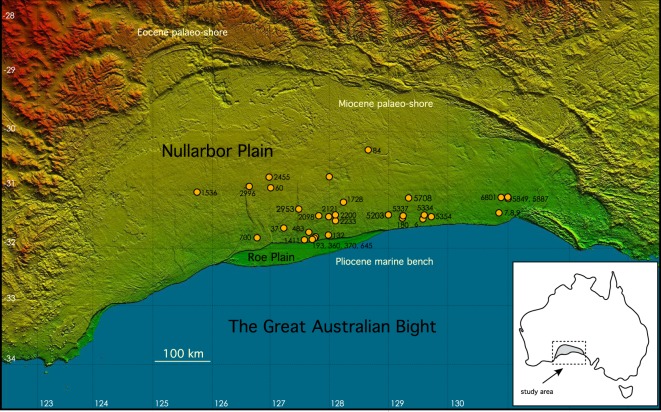
The Nullarbor Plain. Located in southern Australian, this vast, arid limestone plateau constitutes the world’s largest area of exposed karst, and hosts hundreds of shallow caves. Locations marked with a circle and a corresponding number (from the Australian Speleological Federation karst database: https://www.caves.org.au/caves-and-clubs/karst-index-database). Note the Nullarbor ‘6 N’ (Western Australian section) and ‘5 N’ (South Australian section) prefix is omitted for clarity. Caves plotted are those from which speleothem samples have been dated in this study.

**Figure 2:**
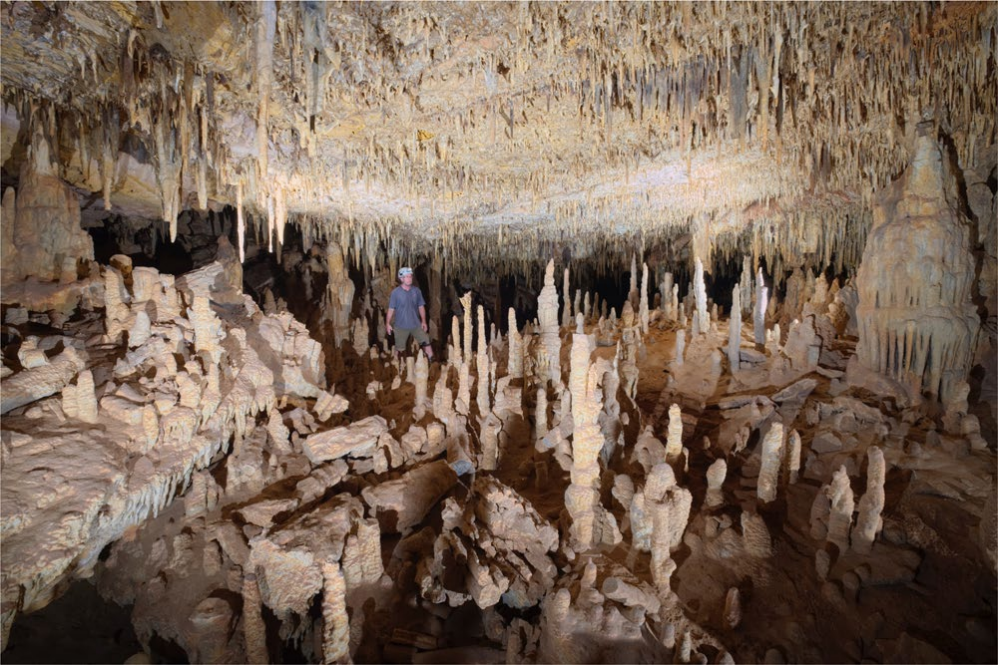
Pliocene cavescapes. The interior of a typical, well-decorated Nullarbor cave. None of these formations are active at the present day and our U-Pb dating program suggests that most of the speleothems are Pliocene in age: these are, in effect, fossil landscapes, frozen in time. These materials, and others like them from around the globe, form a remarkable but largely unrecognized archive of palaeoclimatic and palaeoenvironmental information.

Here, we present 125 U-Pb dates from 31 shallow caves scattered across 100,000 km^2^ of the world’s largest exposed karst terrain, that demonstrate *landscape-scale* preservation of a cave network featuring dense accumulations of mostly Early Pliocene speleothems, including even the most delicate forms (Figs 1 and 2, Supplementary videos 1 and 2). These provide a unique opportunity for documenting the detailed hydroclimatic history of the Pliocene Southern Hemisphere subtropics, but, on a broader level, suggest that preservation of extensive, pre-Quaternary karst provinces might be more frequent than expected, providing large, untapped resources of palaeoenvironmental and palaeoclimatic information.

## Geochronology Results

The age of the enigmatic caves formed beneath the Nullarbor Plain has long been a mystery, in the absence of suitable chronometers (e.g. ref.^14^). The area is currently arid, receiving ~200–250 mm rainfall per year, with potential evapotranspiration of c. 1250 mm/year (ref.^15^) greatly exceeding precipitation, and, with the exception of a thin coastal strip, the region is largely treeless – giving rise to its name. The negative moisture balance, sparse vegetation and lack of a well-developed soil profile on the karst surface inhibit modern speleothem formation and the predominantly shallow caves are dry and dusty, except where they intersect the local water table (Fig. 2, and Supplementary videos)

**Figure 3:**
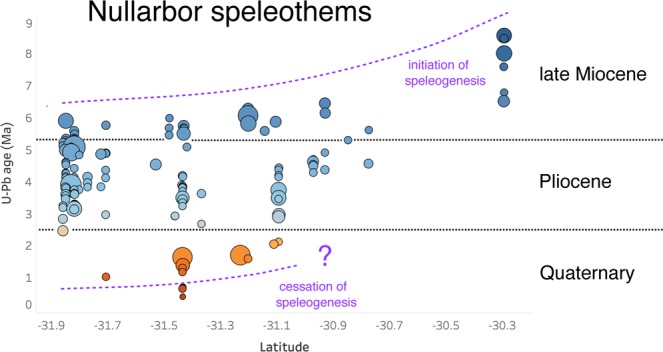
A history of Nullarbor speleogenesis. A compilation of available U-Pb ages, plotted vs. latitude. The size of individual circles indicates the analytical uncertainty on each age determination while the colours code with age – Quaternary samples are orange while Pliocene and late Miocene samples are coloured blue. Dotted lines indicate one possible interpretation of the north-south structure in the data.

Rare, evaporitic calcite, gypsum and halite^16^ speleothems grow episodically beneath occasional drip points and are of Holocene to Late Pleistocene age, but the majority of the formations lie beyond the range of the U-Th geochronometer and thus were, until recently, of unknown age^14^. Our initial exploratory studies, using the U-Pb method to date three speleothems^17,18^, revealed that at least some of the materials from the region were of considerable antiquity – several million years in age. These precursor studies initiated a regional survey of Nullarbor speleogenesis, by far the largest study of its type ever undertaken (see Methods and Supplementary Table 1). By analogy with detrital zircon studies, the number of ages obtained here suggest that the underlying data structure should be adequately represented^19^. Speleothem ages are strongly concentrated within the 5 Ma to 3 Ma window, with very few ages younger than 2.5 million years, or older than 5.5 Ma (Fig. 3). The distribution of ages indicates that dewatering and speleogenesis in the shallow Nullarbor caves (i.e. those hosted in the uppermost stratigraphic unit, the Middle Miocene Nullarbor limestone^20,21^ began no later than the Late Miocene, and that the main phase of speleothem growth occurred during the Early Pliocene, with several individual caves clearly supporting speleothem growth over several million years (Fig. 3). Speleothem growth declined rapidly after 2.5 Ma, near the beginning of the Pleistocene.

**Figure 4:**
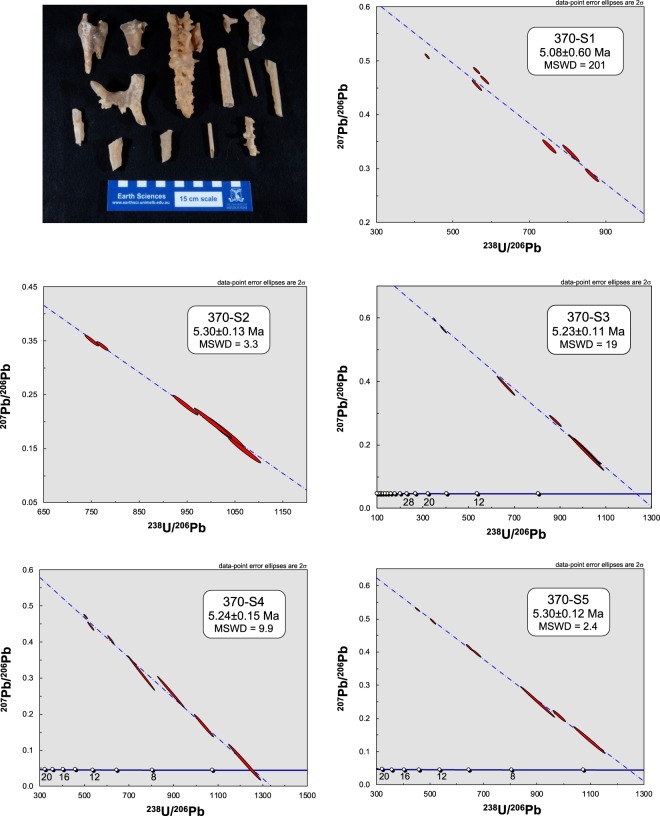
Preservation of straw speleothems. U-Pb isochrons determined on five samples of straw speleothems/helictites from one cave (ASF database 6N-370) illustrating that the preservation of these Pliocene landscapes extends to even the most delicate of formations.

Despite the great age of the speleothems, and the fact that widespread speleothem formation has long since ceased, many Nullarbor caves preserve calcite formations indistinguishable from modern cave systems (Supplementary Fig. S2), including seismically-sensitive features^22^ such as dense ceilings of soda straws and helictites (Fig. 2). In order to test whether these fragile materials were younger than the larger, more robust stalagmites from the same cave, we conducted additional dating of several, randomly sampled, small straw and helictite fragments from one highly decorated ceiling in cave 6N-370. The results (Fig. 4) indicate that this ceiling dates from at least 5 Ma, falling within the range of larger stalagmites from the same cave (3.1–5.6 Ma) and revealing that even these most intricate cave formations are Early Pliocene in age. The inescapable conclusion is that all of the shallow Nullarbor caves were formed in the Miocene-Pliocene and have remained essentially unmodified since that time – ancient worlds, frozen in time. The age and origin of the small number of deeper Nullarbor caves, formed in the underlying Eocene Wilson Bluff^20^ and Oligocene/Early Miocene Abrakurrie^23^ limestones, remain unconstrained by radiometric methods at this time until suitable speleothems can be found for dating.

## Remarkable Preservation of the Nullarbor Caves

It seems that the survival of the Pliocene speleothems of the Nullarbor can be attributed to a coincidence of factors. The Nullarbor Plain itself is a vast ancient seafloor which is assumed to have been uplifted around 14 million years ago^24^ resulting from a general N (down)-SSW (up) tilting of the Australian continent as it drifted north over variations in the local mantle geoid and dynamic topography fields^25^. A broad correlation of speleothem age with latitude (Fig. 3) supports such a hypothesis. This gentle uplift, estimated at 15–20 m Myr^−1^ (ref.^25^), coupled with the considerably wetter climates prevalent during the Early Pliocene^11^ promoted a period of speleothem growth lasting several million years. Starting around 3 Ma, however, and increasing in its intensity around 1 Ma ago, the Australian continent descended into its current, predominantly arid state^11,26^. This aridification likely contributed to the preservation of the Pliocene Nullarbor caves. Since that time the region has experienced relatively little erosion (up to ~4.5 m Ma^−1^, ref.^27^, but possibly much less^28^), and, given the general stability of the continent, probably relatively limited seismic activity. The preservation on the Nullarbor Plain of long fault scarps (>100 km in length) with small vertical offsets, barely visible at ground level and detected using digital elevation models (Fig. 1), is testimony to the long-term stability of the Nullarbor surface^29^. We note that upward migration of caves through roof collapse might be expected to have eliminated, or at least truncated, some shallow Nullarbor caves. However, the hundreds of speleothem-bearing caves on the Nullarbor suggest that, at least in this large, planar arid landscape, such processes have not been sufficiently energetic to destroy the caves, over million-year timescales.

### Speleothems at U-Th equilibrium: the worldwide archive

**Figure 5:**
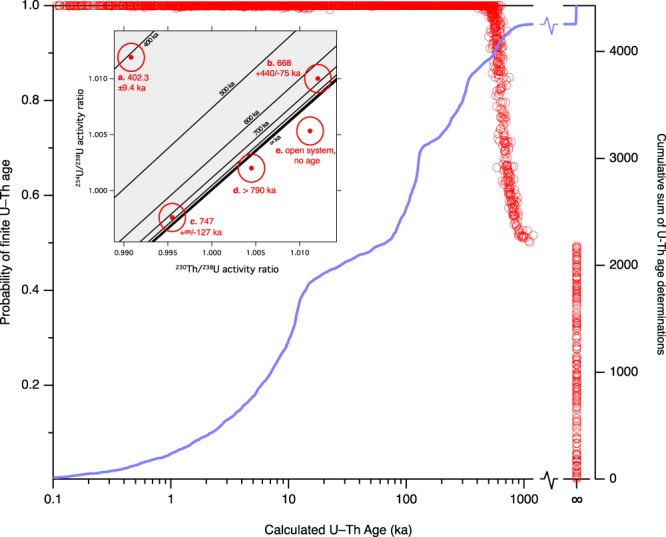
Probability of speleothems returning a finite U-Th age. A body of ca. 4400 clean, closed-system speleothem U-Th analyses from >25 countries, showing their probability of returning a finite age (red circles) and cumulative sum of age determinations (blue line). 275 determinations are within 2σ uncertainty of infinite age and 163 are most likely ‘infinite’, together comprising ca. 10% of the total. *Inset*: example U-Th age determinations in ^230^Th–^234^U-^238^U isochron space (in grey). (**a**) is clearly within U-Th age space, with near-symmetrical uncertainty; (**b**) is near the infinite isochron and gives large asymmetric uncertainty; (**c**) overlaps the infinite isochron but has a > 0.5 probability of being finite; (**d**) partially overlaps U-Th age space and only allows calculation of a minimum age; (**e**) is entirely outside U-Th age space and cannot have behaved as a closed system. All analyses were conducted at the University of Melbourne.

Alternative approaches to dating cave materials, such as cosmogenic burial studies, have demonstrated the Early-Mid Pleistocene to Pliocene antiquity of some cave and palaeokarst systems^30–35^, even in regions that clearly are neither arid, nor characterised by low average denudation rates. In addition, the speleothem palaeoclimate literature itself has always included ‘infinite age’ samples – those beyond the reach of the U-Th decay scheme. This age limit was originally ~300 ka, using alpha particle counting methodologies, but currently, with the latest mass spectrometric methods, now approaches ~640 ka^36^. ‘Infinite age’ speleothems have been reported from most continents (Supplementary Fig. S1) and represent circumstantial evidence for the widespread availability of a terrestrial palaeoclimate archive extending beyond the range of U-Th dating. In order to estimate the potential size of such a resource, we surveyed a set of ca. 4400 U-Th analyses of clean speleothems performed over a period of 15 years in our laboratory^37^, and representing caves situated in ca. 60 karst regions spread across 25 countries. Approximately 10% of these samples, derived from at least 12 countries, have less than 98% probability of returning a finite U-Th age – that is, these samples’ Gaussian 2σ age uncertainties overlap the infinite-age isochron (Fig. 5). Few results from these indeterminate-aged samples have been published, as the U-Th-based studies through which they were discovered have uniformly focussed on younger, U-Th-dateable materials. The potential significance of such samples has therefore rarely been considered. The ca. 10% of our U-Th analyses that are ‘infinite’ underestimates the proportion of undateable specimens as we have tended to focus our U-Th efforts on settings found to have younger speleothems.

For entire cave provinces to persist well beyond 1 Ma may have required an interruption to the prevalent hydrological cycle – in the case of the Nullarbor, Australia’s drift into aridity within the last few million years. In other areas where extensive occurrences of ancient speleothems have been noted (e.g. Yukon, Baikal) the development of permafrost may have been pivotal in their survival. Such circumstances are probably rare. The majority of published ‘infinite’ age speleothems, instead, appear to represent the attenuated end of a spectrum of speleothem ages within active cave provinces that are dominated by younger samples. In many cases, long-lived cave systems may continue producing speleothems for millions of years thus preserving a near-continuous climatic archive: caves in the European Alps provide a good example, with U-Th and U-Pb ages^7,8,38^ demonstrating speleothem growth from at least 2 Ma up to the present.

There are of course impediments to the palaeoclimatic interpretation of speleothem data across large temporal and spatial scales. In particular, individual samples only record relatively short periods of time and traditional speleothem climate proxies such as oxygen isotopes experience site-specific effects. Nevertheless, there is abundant potential for producing relatively long records documenting the timing of glacial-interglacial shifts^7^ or the nature of landscape change through alternative proxies such as speleothem palynology^39^.The U-Pb methodologies described above, and deployed for the first time at a regional scale in this study, provide an important new avenue for exploring the longevity of caves in the landscape and the preservation of speleothems as climate archives. Our results suggest that, even at a regional scale, caves such as those studied here can have lifetimes measured in many millions of years and survive for similar timescales after cessation of speleothem growth. Carbonates make up an estimated ~30% of the mass of Phanerozoic sediments^40^, and karst phenomena have been present on the Earth throughout that time, possibly with recognisably ‘modern’ rates of limestone dissolution and calcite precipitation established as a response to the rise of terrestrial vegetation in the Silurian^30^. Given this, the prospects for finding other speleothem archives older than ~600 ka, concealing undocumented Neogene climate histories remains high. Once cave formation transitions from an erosional void-creating process to an accumulative void-filling process, these underground spaces and the speleothems formed in them may have much to tell us about ancient climates and landscapes.

## Methods

The analytical methods employed in this study follow closely those published previously^12,13,17^. Nullarbor speleothem samples typically maintain well preserved laminations and are often indistinguishable from Holocene samples (Supplementary Fig. S2). Occasional domains appear to contain recrystallised calcite but, even in these, U-Pb systematics are preserved, suggesting minimal, if any, elemental redistribution. This is consistent with our findings from well-preserved Permian speleothem samples which are much older (300 Ma) and yet which preserve their U-Pb and trace element distributions^13^. Multiple aliquots, typically weighing ~50 mg, were removed from each speleothem sample using a dental drill. The pieces of calcite were then placed into pre-cleaned disposable polyethylene cups and moved to a multiple-HEPA filtered clean room environment. Samples were briefly leached 2 times in very dilute (~0.01 M) three-times teflon distilled HCl, with each cycle lasting around a minute, and then repeatedly washed in ultra-pure water before being dried in a HEPA filtered laminar flow hood. This step is critical to the elimination of Pb contaminants resulting from sample handling which can easily dominate the Pb budget of the entire sample unless removed.

Individual samples were weighed into pre-cleaned teflon beakers and treated with sufficient 6 N HCl to ensure complete dissolution. A mixed ^233^U-^205^Pb tracer, calibrated against EarthTime (http://www.earth-time.org) reference solutions, was then weighed into the vials and each one sealed and refluxed on the hotplate for several hours to ensure complete sample-spike equilibration. Samples were then dried down and taken up in 0.6 N HBr for Pb separation using a single pass over AG 1X-8 anion exchange resin. The eluate from the first column (U + matrix) was subsequently processed through the same column now emptied of AG1X-8 and refilled with Eichrom TRU ion-specific resin, to separate U. Pb blanks were typically 10 ± 5 pg and were corrected for. U blanks were insignificant relative to the amounts of U being processed.

Isotope ratios were determined on a Nu Instruments *Plasma* MC-ICPMS using a DSN-100 desolvation unit and MicroMist glass nebuliser, operating in the range 50–100 µl/min uptake. Instrumental mass bias effects were monitored and corrected using NIST SRM 981 reference material in the case of Pb, and the sample’s internal (=137.88) ^238^U/^235^U ratio in the case of U. Instrument data files were processed initially using an in-house designed importer, operating within the *Iolite* software environment^41^, which considers all data and reference material analyses obtained throughout a particular analytical session and permits a variety of corrections for instrumental mass bias and drift. The resulting data, now corrected for instrumental effects, were then blank corrected and isotope-dilution calculations performed using the Schmitz and Schoene^42^ software.

^238^U/^206^Pb-^207^Pb/^206^Pb isochron regressions were calculated using ‘Isoplot Ex’^43^ and are labelled ‘1’ or ‘2’ in Supplementary Table 1, corresponding to the well-known Model 1 and Model 2 isochron types distinguished by MSWD less than or greater than 2.5 respectively. In the former it is assumed that the assigned analytical errors are the only source of data scatter and points are therefore weighted according to the inverse square of these uncertainties. In situations where the software detects a low probability of fit based upon assigned errors alone (i.e. there is likely additional *geological* scatter) a so-called ‘Model 2’ fit is employed which instead assigns equal weights and zero error-correlations to each point. From a philosophical standpoint there are many reasons to move away from such ‘stepwise’ changes in uncertainty handling and some attempts have been made by the geochronology community to move in this direction (e.g. ref.^44^). As yet, however, these robust statistical methods are yet to be implemented for the U-Pb system and so the traditional approach (and that invoked by Isoplot Ex) is employed in this study. Note that the findings of this study are in no way influenced by these subtle analytical nuances.

Initially, all age determinations from this study consisted of multiple sample aliquots (and many remain of this type). It quickly became apparent from these data, however, that the composition of the common Pb component incorporated into Nullarbor speleothems was relatively constant across large geographic distances (the value used here is ^207^Pb/^206^Pb = 0.824 with 2 sigma relative uncertainty of 8.9%). As discussed in detail by Woodhead *et al*.^45^, this then allows the use of a regional common Pb composition in anchoring isochrons if required, e.g. where there is insufficient spread in U/Pb ratios within a given speleothem. This is a commonly used approach in U-Pb dating of samples with appreciable quantities of common Pb, e.g. perovskite^46^ and we believe that it is similarly ‘fit for purpose’ in a large-scale reconnaissance geochronology survey such as this. In situations where a regional common Pb value has been utilized in isochron construction the suffix ‘A’ is added to the Isochron type in Supplementary Table 1. We stress, however, that all age determinations in this study are calculated from at least three data points (one of which may be the regional common Pb composition). As a result, all ages are considered accurate within the uncertainties provided. For a general discussion of the accuracy of our U-Pb results readers are referred to Woodhead and Pickering^47^, Section 2.4.

Isochron ages were obtained using in-house software and an iterative procedure to calculate the intersection of isochrons with an appropriate disequilibrium Concordia and assuming negligible initial ^230^Th (^230^Th/^232^Th activity ratios are typically in excess of 5,000 but range up to ~50,000 (ref.^17^). Any inaccuracies in final U-Pb age determinations caused by the assumption of zero ^230^Th only amount to ~10 kyr maximum and thus have minimal impact on the final uncertainty budget. There is no straightforward means of assessing the likely ^234^U/^238^U initial activity ratios of Nullarbor cave dripwaters and thus it is impossible to make a robust correction for initial disequilibrium effects. We therefore base our assessment on the only materials that we have found (in many years of searching) from the Nullarbor which retain measurable ^234^U disequilibrium – a total of 13 Pleistocene samples. These show no apparent change in ^234^U with age and provide a median ^234^U/^238^U initial of 1.00 with a 2 SD of 0.26 (Supplementary Table 3; refs^17,48^). Based upon these data we impose a necessarily broad range in initial activity ratio of 1 ± 0.3 during calculation of disequilibrium-corrected ages. For Nullarbor samples, the correction for initial disequilibrium in ^230^Th results in an increase in age of ~100 kyr while the correction for initial disequilibrium in ^234^U does not change the calculated age but results in an approximate doubling of the age uncertainty.

In order to estimate what proportion of speleothems submitted for U-Th dating return ‘infinite’ ages beyond the analytical range of U-Th, we extracted a subset of 4400 clean (i.e with low detrital Th contributions) and precise age results from a body of ca. 13,000 U-Th analyses conducted at the University of Melbourne from 2003 to 2018, analysed following previously published methods^37^.

For Fig. 2, which includes an image of one of the authors, informed consent was obtained for open-access publication.

## Supplementary information

Supplementary information

## Data Availability

All data generated or analysed during this study are included in this published article (and its Supplementary Information files).
