# Supplementary material for: The antiquity of Nullarbor speleothems and implications for karst palaeoclimate archives
Source: Sci Rep. 2019 Jan 24;9:603. doi: 10.1038/s41598-018-37097-2 (PMC6345804; doi:10.1038/s41598-018-37097-2)
Supplement: Supplementary file 1 — Supplementary information [file 41598_2018_37097_MOESM1_ESM.pdf]

The antiquity of Nullarbor speleothems and implications for karst palaeoclimate archives

Authors: Jon D. Woodhead[1*], J.M. Kale Sniderman[1], John Hellstrom[1], Russell N. Drysdale[2,3], Roland Maas[1], Nicholas White[4], Susan White[4,5], Paul Devine[6]

Affiliations:

[1] School of Earth Sciences, University of Melbourne, Parkville, VIC 3010, Australia.

[2] School of Geography, University of Melbourne, Parkville, VIC 3010, Australia.

[3] Environnements, Dynamiques et Territoires de la Montagne, UMR CNRS, Université de Savoie-Mont Blanc, 73376 Le Bourget du Lac, France.

[4] Victorian Speleological Association, GPO Box 5425, Melbourne, VIC 3001, Australia

[5] School of Life Sciences, La Trobe University, Bundoora, VIC 3086, Australia

[6] (deceased) Speleological Research Group of Western Australia, P.O. Box 1611, East Victoria Park, WA 6981, Australia

*corresponding author : jdwood@unimelb.edu.au

Supplementary Information

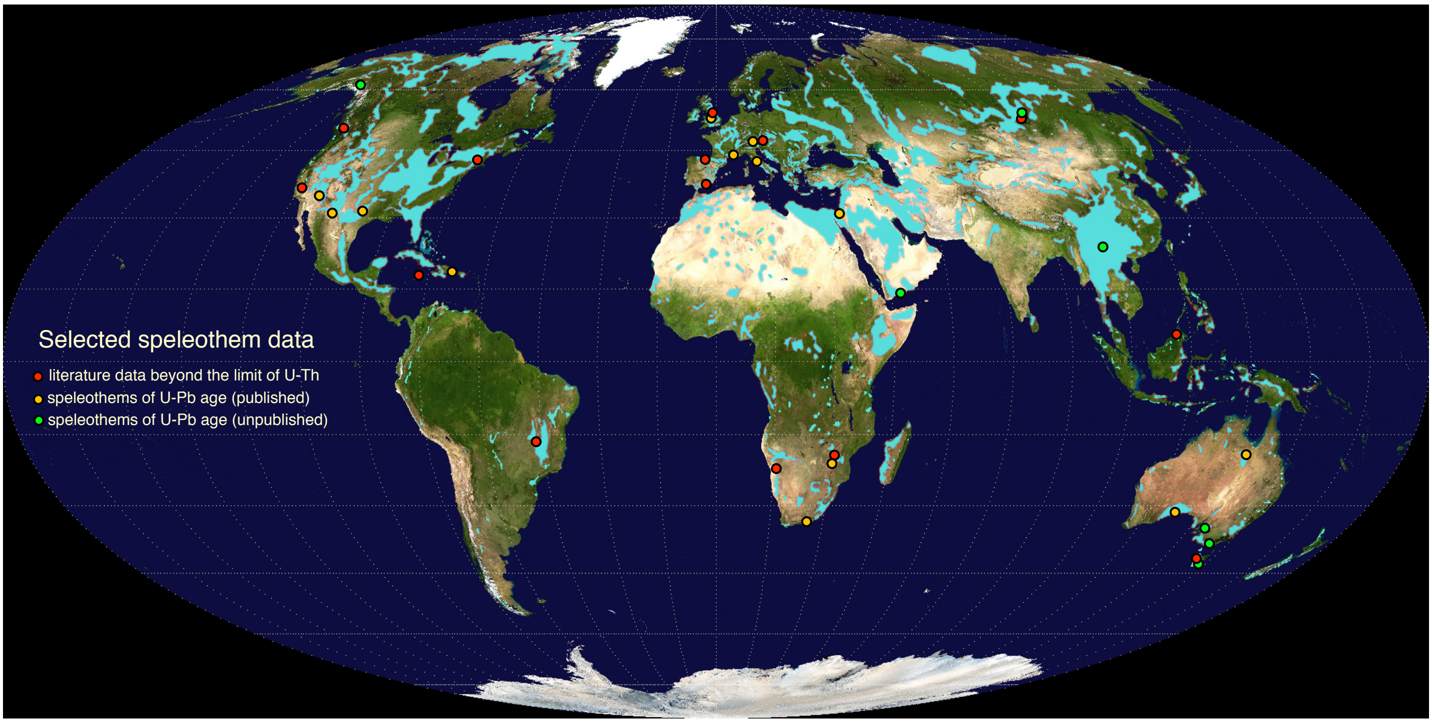

Supplementary Figure S1

Selected locations of speleothems known to be beyond the range of the U-Th chronometer. The underlying map of global karst distribution was sourced from Wikimedia Commons (author:ulrichstill, https://commons.wikimedia.org/wiki/File:Carbonate-outcrops_world.jpg and licensed under the CC BY-SA DE license: https://creativecommons.org/licenses/by-sa/2.0/de/deed.en). The colour of the carbonate outcrops on the original map was changed to light blue and then our speleothem location data plotted on the map. Speleothem data sources: **Literature data noted to be beyond the utility of the U-Th chronometer** (Auler, 2006; Bischoff, 2003; Dirks, 2017; Geyh, 2014; Gibert, 2016; Goede, 1983 ; Latham, 1982; Lauritzen, 2000; Leél-Össy, 2011; Lundberg, 2010 ; MacPhee, 1989; Moseley, 2013 ; Stock, 2005; Vaks, 2013), **published speleothem U-Pb studies** (Bajo et al., 2012; Cliff et al., 2010; Dertnig et al., 2017; Lundberg et al., 2000; Michel et al., 2017; Rosenberger et al, 2015; Pickering et al., 2013; Polyak et al., 2008; Richards et al., 1998; Sniderman et al., 2016; Vaks et al., 2013; Woodhead et al., 2010; Woodhead et al., 2016). **Unpublished speleothem U-Pb studies** are all from the University of Melbourne laboratory (Woodhead et al., unpublished data). See below for references.

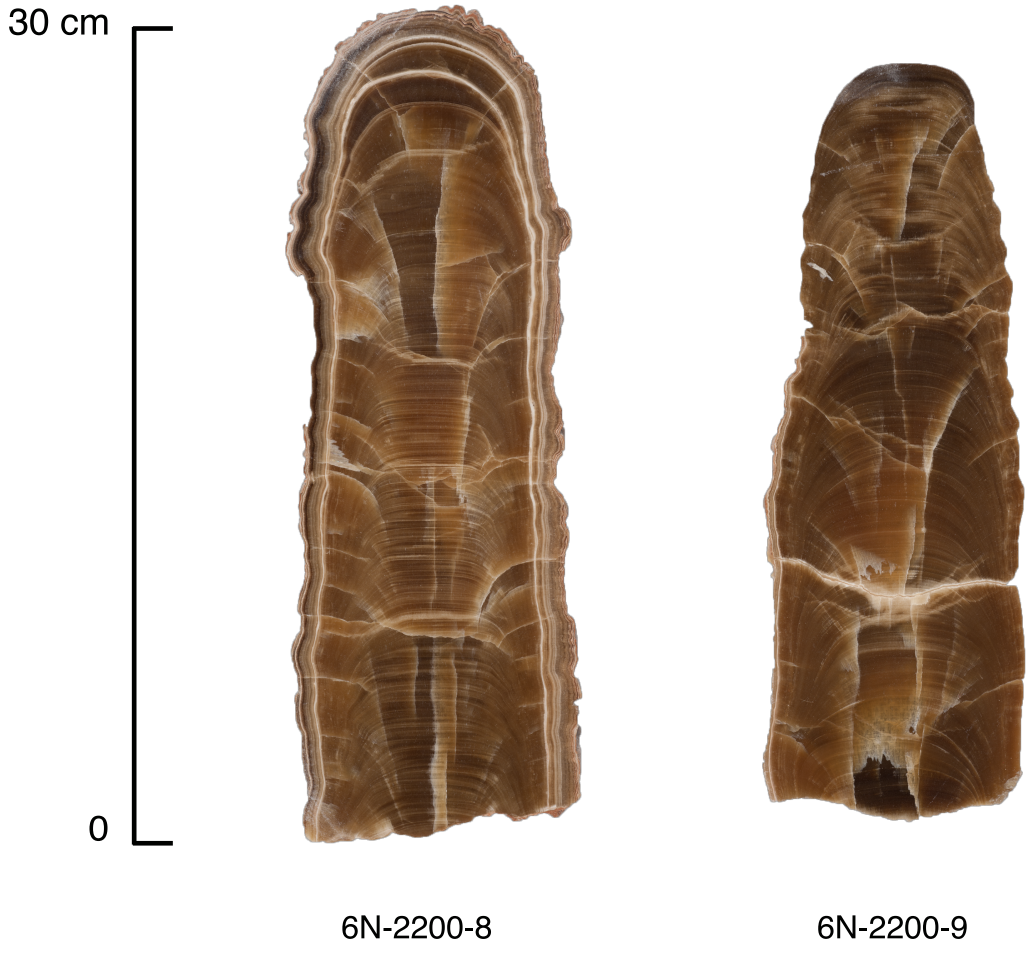

Supplementary Figure S2
Typical Pliocene Nullarbor speleothems exhibiting fine-scale growth laminae identical to modern spelothems. Many of the Nullarbor samples are dark in colour – a feature usually attributed to their high organic content. Very few samples show evidence of recrystallisation.

The following two videos are hosted on 'figshare' and links are provided.

Supplementary video V1 A typical well decorated Nullarbor cave, normal video
https://doi.org/10.26188/5becb576e6616

Supplementary video V2 A typical well decorated Nullarbor cave, 3D anaglyph video (requires blue-red glasses for optimal 3D viewing)
https://doi.org/10.26188/5becb9ebdabbd

References for Supplementary material

Auler, A.S. *et al.* U-series dating and taphonomy of Quaternary vertebrates from Brazilian caves. *Palaeogeography, Palaeoclimatology, Palaeoecology* **240**, 508-522 (2006).

Bajo, P., Drysdale, R., Woodhead, J., Hellstrom, J. & Zanchetta, G. High-resolution U-Pb dating of an early Pleistocene stalagmite from Corchia Cave (central Italy). *Quaternary Geochronology* **14**, 5-17 (2012).

Bischoff, J.L. *et al.* The Sima de los Huesos hominids date to beyond U/Th equilibrium (>350ka) and perhaps to 400-500 kyr: new radiometric dates. *Journal of Archaeological Science* **30**, 275-280 (2003).

Cheng, H., *et al.,* Improvements in ^{230}Th dating, ^{230}Th and ^{234}U half-life values, and U–Th isotopic measurements by multi-collector inductively coupled plasma mass spectrometry. *Earth and Planetary Science Letters* **371–372**, 82–91 (2013).

Cliff, R.A., Spötl, C. & Mangini, A. U-Pb dating of speleothems from Spannagel cave, Austrian Alps: a high-resolution comparison with U-Series ages. *Quaternary Geochronology* **5**, 452-458 (2010).

Dertnig, F., Stüwe, K., Woodhead, J., Stuart, F.M.& Spötl, C. Constraints on the Miocene landscape evolution of the Eastern Alps from the Kalkspitze region, Niedere Tauern (Austria) *Geomorphology* **299**, 24-38 (2017).

Dirks, P.H.G.M *et al.* The age of *Homo naledi* and associated sediments in the Rising Star Cave, South Africa. *eLife* 2017;6:e24231 (2017).

Geyh, M.A. & Heine, K. Several distinct wet periods since 420ka in the Namib Desert inferred from U-series dates of speleothems. *Quaternary Research* **81**, 381-391 (2014).

Gibert, L. *et al.* Chronology for the Cueva Victoria fossil site (SE Spain): evidence for early Pleistocene Afro-Iberian dispersals. *Journal of Human Evolution* **90**, 183-197 (2016).

Goede, A. & Harmon, R.S. Radiometric dating of Tasmanian speleothems – evidence of cave evolution and climate change. *Journal of the Geological Society of Australia* **30**, 89-100 (1983).

Hellstrom, J., U–Th dating of speleothems with high initial ^{230}Th using stratigraphical constraint. *Quaternary Geochronology* **1**, 289–295 (2006).

Latham, A., Schwartz, H.P., Ford, D.C. & Pearce, G.W. The paleomagnetism and U-Th dating of three Canadian speleothems: evidence for the westward drift, 5.4-2.1 ka BP. *Canadian Journal of Earth Sciences* **19**, 1985-1995 (1982).

Lauritzen, S-E. & Mylroie, J.E. Results of a speleothem U/Th dating reconnaissance from the Helderberg Plateau, New York. *Journal of Cave and Karst Studies* **62**, 20-26 (2000).

Leél-Össy,S., Szanyi, G. & Surányi, G. Minerals and speleothems of the Jósef-hegy cave (Budapest, Hungary). *International Journal of Speleology* **40**, 191-203 (2011).

Lundberg, J., Ford, D.C. & Hill, C.A. A preliminary U-Pb date on cave spar, Big Canyon, Guadalupe Mountains, New Mexico. *Journal of Cave and Karst Studies* ***62*****,** 144-148 (2000).

Lundberg, J., Lord, T.C. & Murphy, P.J. Thermal ionization mass spectrometer U-Th dates on Pleistocene speleothems from Victoria Cave, North Yorkshire, UK: Implications for palaeoenvironment and stratigraphy over multiple glacial cycles. *Geosphere* **6**, 379-395 (2010).

MacPhee, R.D.E., Ford, D.C. & McFarlane, D.A. Pre-Wisconsinan mammals from Jamaica and Models of Late Quaternary extinction in the Greater Antilles. *Quaternary Research* **31**, 94-106 (1989).

Michel, V *et al.* New evidence of the early presence of hominids in Southern Europe. *Scientific Reports*. **7**, 10074 (2017).

Moseley, G.E. *et al.* U-Th dating of speleothems to investigate the evolution of limestone caves in the Gunung Mulu National Park, Sarawak, Malaysia. *Cave and Karst Science* **40**, 13-16 (2013).

Rosenberger, A.L. *et al.* 1.32±0.11 Ma age for underwater remains constrain antiquity and longevity of the Dominican primate Antillothrix bernensis. *Journal of Human evolution* **88**, 85-96 (2015).

Pickering, R. *et al.* Palaeoanthropologically significant South African Sea Caves dated to 1.0 Million years using a combination of U-Pb, TT-OSL and palaeomagnetism. *Quaternary Science Reviews* **65**, 39-52 (2013).

Polyak, V., Hill, C. & Asmerom, Y. Age and evolution of the Grand Canyon revealed by U-Pb dating of water table type speleothems. *Science* **319**, 1377-1380 (2008).

Richards, D.A., Bottrell, S.H., Cliff, R.A., Strohle, K., Rowe, P.J. U-Pb dating of a speleothem of Quaternary age. *Geochimica et Cosmochimica Acta* **62**, 3683-3688 (1998).

Sniderman, J.M.K. *et al.* Pliocene reversal of late Neogene aridification. *Proceedings of the National Academy of Sciences* **113**, 1999-2004 (2016).

Stock, G.M., Granger, D.E., Sasowsky, I.D., Anderson, R.S. & Finkel, R.C., Comparison of U-Th, paleomagnetism, and cosmogenic burial methods for dating caves: Implications for landscape evolution studies. *Earth and Planetary Science Letters*, **236**, 388–403 (2005).

Vaks, A. *et al.* Pliocene-Pleistocene climate of the northern margin of Saharan-Arabian Desert recorded in speleothems from the Negev Desert, Israel. *Earth and Planetary Science Letters* **368**, 88-100 (2013).

Vaks, A. *et al.* Speleothems reveal 500,000-year history of Siberian permafrost. *Science* **340**, 183-186 (2013).

Woodhead, J. *et al.* Developing a radiometrically-dated chronologic sequence for Neogene biotic change in Australia, from the Riversleigh World Heritage Area of Queensland. *Gondwana Research* **29**, 153-167 (2016).

Woodhead, J. *et al.* Speleothem climate records from deep time? Exploring the potential with an example from the Permian. *Geology*, **38**, 455-458 (2010).

Supplementary Table 1. U-Pb isotopic and age data for Nullarbor speleothems.

Bold type indicates cave number as assigned by Australian Speleological Federation. Normal type indicates sample ID where more than one sample derives from an individual cave.

Ages are corrected for initial disequilibrium in the decay chain using 234/238i = 1±0.3.

**Cave number** sample ID	**U ppm**	**Pb ppm**	**$^{238}U^{206}Pb$**	**% err**	**$^{207}Pb^{206}Pb$**	**% err**	**Corr. coeff.**	**Isochron type**	**MSWD**	**Age (Ma)**	**uncertainty 2σ**
**5N-6**											
3.1	0.495	0.0006	1034.2	5.7	0.0915	51.08	-0.9992	1A	0.84	**5.988**	**0.114**
	0.476	0.0010	851.4	9.2	0.2200	1.00	-0.9896				
	0.476	0.0005	1076.9	7.7	0.0539	121.70	-0.9938				
**5N-7**											
MW-1	2.152	0.0020	1825.8	2.9	0.2535	7.46	-0.9922	2A	5.7	**2.689**	**0.113**
	2.102	0.0018	1880.3	3.0	0.2371	8.54	-0.9995				
	2.188	0.0021	1781.6	2.2	0.2741	5.06	-0.9936				
	2.200	0.0018	1929.7	2.2	0.2246	6.57	-0.9993				
MW-2	1.401	0.0009	1814.7	5.1	0.0557	78.52	-0.9999	2	14	**3.625**	**0.151**
	1.465	0.0008	1860.7	2.5	0.0390	56.37	-0.9992				
	1.465	0.0012	1621.3	1.9	0.1471	9.97	-0.9966				
**6N-37**											
MLL	1.329	0.0121	321.8	0.2	0.6777	0.12	-0.8219	1A	1.9	**4.866**	**0.204**
	1.414	0.0142	296.1	0.2	0.6934	0.11	-0.8172				
**6N-60**											
60-14-16	0.738	0.0008	1232.1	2.6	0.1673	11.50	-0.9982	1A	0.58	**4.555**	**0.115**
	0.685	0.0011	1062.2	1.9	0.2618	4.76	-0.9986				
60-14-15	0.606	0.0030	521.0	2.1	0.5500	1.35	-0.9992	1	0.26	**4.295**	**0.128**
	0.604	0.0019	734.6	2.8	0.4451	2.87	-0.9991				
	0.608	0.0012	994.8	6.4	0.3148	12.07	-0.9999				
60-14-17	0.227	0.0006	813.7	4.5	0.3683	6.59	-0.9998	1A	0.7	**4.628**	**0.244**
	0.229	0.0006	830.6	5.1	0.3615	7.68	-0.9997				
	0.225	0.0007	736.3	5.3	0.4112	6.40	-0.9992				
	0.214	0.0007	742.2	9.8	0.4007	12.49	-0.9999				
60-14-10	0.823	0.0008	1282.5	3.1	0.1144	21.53	-0.9992	1A	0.54	**4.708**	**0.116**
	0.866	0.0010	1193.4	4.0	0.1660	18.05	-0.9994				
60-14-18	0.574	0.0006	1263.2	3.8	0.1545	18.60	-0.9907	1A	0.13	**4.497**	**0.156**
	0.581	0.0005	1373.2	5.6	0.0828	55.70	-0.9998				
**6N-84**											
84-01	0.149	0.0011	318.8	1.4	0.5211	1.05	-0.9991	2	26	**8.483**	**0.142**
	0.149	0.0011	328.2	1.7	0.5154	1.32	-0.9973				
	0.120	0.0037	102.6	0.9	0.7517	0.19	-0.9929				
	0.132	0.0003	671.4	3.5	0.1498	16.99	-0.9565				
84-FSCR02	0.612	0.0009	872.2	1.0	0.1422	5.22	-0.9396	1A	0.36	**6.562**	**0.118**
	0.553	0.0016	633.8	1.0	0.3225	1.83	-0.9961				
84-5	0.124	0.0093	44.7	0.5	0.8009	0.12	-0.7044	2	8.2	**8.02**	**0.462**
	0.122	0.0154	26.9	0.4	0.8195	0.11	-0.6034				
	0.184	0.0037	153.7	3.3	0.6954	1.02	-0.9931				
	0.129	0.0059	71.7	1.2	0.7741	0.25	-0.2908				
	0.136	0.0050	89.1	1.4	0.7546	0.31	-0.9598				
84-9	0.519	0.0009	802.2	1.8	0.1905	6.81	-0.9810	1	0.65	**6.796**	**0.112**
	0.941	0.0017	795.7	1.0	0.1957	3.48	-0.9981				
	0.920	0.0021	719.5	1.3	0.2664	1.92	-0.7751				
	0.930	0.0022	705.9	0.8	0.2756	1.94	-0.9959				
84-BL1	0.046	0.0019	80.8	2.7	0.7483	0.60	-0.9889	1A	0.83	**8.583**	**0.423**
	0.048	0.0049	33.0	1.3	0.7977	0.21	-0.9056				
84-BL4	0.205	0.0016	325.0	2.1	0.5143	1.66	-0.9528	1	2.8	**7.597**	**0.123**

	0.146	0.0094	49.8	0.4	0.7526	0.14	-0.7590				
	0.188	0.0012	359.6	2.4	0.4774	2.29	-0.9177				
	0.199	0.0016	305.9	1.9	0.5301	1.35	-0.9988				
**6N-132**											
132-14-33	0.558	0.0011	989.5	1.3	0.3868	1.76	-0.9961	1	3	**3.831**	**0.113**
	0.536	0.0006	1387.4	3.5	0.2027	12.22	-0.9993				
	0.473	0.0005	1384.4	3.5	0.2140	11.52	-0.9427				
	0.522	0.0005	1436.2	3.0	0.1827	11.97	-0.9969				
**5N-180**											
5.4	1.045	0.0011	1641.1	2.6	0.2715	6.16	-0.9969	1A	0.38	**2.93**	**0.121**
	0.919	0.0012	1461.6	3.0	0.3347	5.16	-0.9843				
**6N-193**											
193-1	0.636	0.0020	673.6	1.9	0.4140	2.21	-0.9978	2	4.1	**5.092**	**0.121**
	0.624	0.0079	232.3	0.4	0.6758	0.17	-0.8885				
	0.504	0.0104	149.4	0.3	0.7241	0.12	-0.8052				
	0.499	0.0072	207.8	0.4	0.6909	0.15	-0.8713				
193-2	0.380	0.0005	1103.4	5.1	0.1777	21.00	-0.9950	2A	5	**4.917**	**0.571**
	0.535	0.0011	920.6	4.3	0.2989	8.85	-0.9994				
193-3	1.329	0.0012	1470.0	2.1	0.1392	11.44	-0.9959			**3.915**	**0.814**
	1.085	0.0013	1303.1	0.9	0.2406	2.63	-0.8553	2A	26		
**6N-360**											
36-14-12	0.733	0.0131	176.5	0.3	0.7444	0.12	-0.7753	1	0.37	**3.762**	**0.115**
	0.712	0.0058	357.0	0.6	0.6648	0.25	-0.9605				
	0.755	0.0046	451.8	0.7	0.6235	0.32	-0.9807				
**6N-370**											
370 1	0.815	0.0047	487.6	1.0	0.6275	0.47	-0.9900	1	9	**3.63**	**0.17**
	0.777	0.0087	273.0	0.6	0.7166	1.00	-0.9900				
	0.890	0.0152	186.2	2.0	0.7588	1.18	-0.0256				
370-3A	0.692	0.0006	1668.8	4.5	0.2029	15.48	-0.9992	1	0.89	**3.202**	**0.113**
	0.715	0.0008	1547.4	4.2	0.2465	11.31	-0.9991				
	0.683	0.0010	1301.9	3.3	0.3416	5.40	-0.9996				
	0.698	0.0006	1761.2	3.5	0.1688	15.48	-0.9995				
370-3B	0.499	0.0037	396.3	0.9	0.6584	0.36	-0.9711	2	12	**3.62**	**0.135**
	0.640	0.0086	230.2	0.5	0.7289	0.15	-0.8175				
	0.435	0.0036	360.8	1.1	0.6714	0.39	-0.9864				
	0.627	0.0021	728.6	2.2	0.5166	1.66	-0.9983				
	0.427	0.0049	266.0	0.9	0.7151	0.30	-0.8012				
	0.681	0.0033	553.7	1.3	0.5940	0.57	-0.9900				
370-5	0.923	0.0026	830.0	3.9	0.4670	3.84	-0.9407	1	1.1	**3.76**	**0.12**
	0.930	0.0013	1297.2	4.9	0.2553	12.66	-0.9932				
	0.945	0.0027	834.7	5.1	0.4611	5.00	-0.9963				
	0.943	0.0009	1548.3	7.9	0.1408	43.34	-0.9958				
	0.910	0.0013	1319.7	8.7	0.2490	23.19	-0.9999				
370-7	0.385	0.0022	487.9	1.9	0.6220	0.88	-0.9993	1	1.6	**3.167**	**0.138**
	0.408	0.0024	485.7	1.2	0.6236	0.55	-0.9990				
	0.493	0.0023	591.5	1.9	0.5836	1.05	-0.9998				
	0.452	0.0024	519.5	1.1	0.6110	0.54	-0.9998				
	0.455	0.0026	497.9	1.5	0.6173	0.70	-0.9995				
	0.446	0.0036	366.9	1.7	0.6641	0.63	-0.9997				
370 9	0.736	0.0005	1764.5	3.3	0.0773	35.40	-0.9977	2A	5.3	**3.632**	**0.135**
	0.754	0.0004	1892.4	6.1	0.0155	351.00	-0.9995				
370-11	2.232	0.0016	1474.2	1.1	0.0823	11.50	-0.9722	1	0.65	**4.149**	**0.118**
	2.213	0.0018	1401.4	0.8	0.1082	5.62	-0.9632				
	2.247	0.0015	1536.0	1.0	0.0661	13.13	-0.9872				
	2.233	0.0017	1446.4	1.1	0.1025	8.72	-0.9515				
	2.575	0.0016	1563.2	0.9	0.0573	13.78	-0.9934				
	2.651	0.0021	1435.1	1.8	0.0950	15.21	-0.9977				
370-12	0.711	0.0016	1011.8	2.5	0.4464	2.61	-0.9607	1A	0.63	**3.323**	**0.113**
	0.765	0.0008	1600.1	4.0	0.2097	13.44	-0.9997				
370-13	0.805	0.8010	3.4	0.1	0.8081	2.41	-0.9980	1A	0.068	**4.414**	**0.12**
	2.120	0.0019	1337.2	0.7	0.1291	4.45	-0.9890				

370-16	0.494	0.0006	1160.4	3.4	0.1409	18.69	-0.9995	2	25	**4.973**	**0.119**
	0.446	0.0004	1248.6	6.1	0.0852	59.29	-0.9996				
	0.468	0.0005	1234.0	5.0	0.0916	44.85	-0.9973				
	0.477	0.0012	792.7	2.7	0.3446	4.35	-0.9950				
	0.385	0.0012	680.7	1.9	0.4164	2.33	-0.9498				
	0.404	0.0021	490.0	1.4	0.5347	1.00	-0.9979				
	0.376	0.0016	567.0	1.5	0.4804	1.33	-0.9973				
370-17	1.832	0.0354	158.8	0.2	0.7158	0.11	-0.8208	2	2.7	**5.337**	**0.117**
	1.657	0.0317	159.8	0.1	0.7140	0.10	-0.8377				
	1.087	0.0200	165.9	0.2	0.7105	0.11	-0.8202				
	0.423	0.0019	523.7	1.9	0.4880	1.65	-0.9994				
	3.090	0.0509	183.1	0.2	0.7002	0.11	-0.8373				
370-19	2.063	0.0390	162.2	0.2	0.7154	0.11	-0.7858	1	0.017	**5.588**	**0.155**
	2.050	0.0309	198.4	0.2	0.6916	0.11	-0.7885				
	1.832	0.0333	168.0	0.2	0.7117	0.11	-0.5780				
	1.591	0.0337	146.1	0.3	0.7261	0.12	-0.7900				
	2.227	0.0363	185.1	0.2	0.7003	0.11	-0.8151				
**6N-483**											
483-1	0.793	0.0028	898.7	3.6	0.7383	1.17	-0.6265	1A	0.22	**1.039**	**0.109**
	0.781	0.0010	2195.4	5.7	0.5898	3.08	-0.9994				
483-2	0.276	0.0026	302.0	0.6	0.6432	0.26	-0.9990	1	0.25	**4.899**	**0.145**
	0.291	0.0028	300.7	1.3	0.6436	0.54	-0.9967				
	0.181	0.0038	148.3	0.9	0.7310	0.29	-0.1330				
483-WM04	1.598	0.0034	908.5	1.6	0.3565	2.47	-0.9973	2A	5.9	**4.359**	**0.13**
	1.080	0.0023	921.4	1.4	0.3525	2.21	-0.9989				
	1.031	0.0033	717.4	1.4	0.4598	1.39	-0.9763				
483-6	1.218	0.0014	1509.2	1.9	0.2824	4.28	-0.9995	1A	3.5	**2.974**	**0.129**
	1.326	0.0013	1647.8	2.3	0.2403	6.44	-0.9973				
483-7	0.735	0.0027	658.8	0.9	0.4974	0.74	-0.9984	2	18	**4.126**	**0.115**
	0.825	0.0036	572.1	1.1	0.5372	0.75	-0.9995				
	0.781	0.0027	689.0	1.4	0.4830	1.21	-0.9986				
	1.087	0.0065	450.9	0.3	0.5952	0.18	-0.9942				
	0.965	0.0057	456.5	0.8	0.5952	0.44	-0.9979				
	0.980	0.0051	503.1	0.4	0.5702	0.26	-0.9986				
	1.055	0.0080	370.6	0.3	0.6348	0.11	-0.9990				
	1.038	0.0075	387.3	0.5	0.6252	0.23	-0.9964				
	0.985	0.0048	529.1	0.7	0.5614	0.45	-0.9666				
	0.987	0.0014	1160.9	2.1	0.2579	5.20	-0.9977				
483-9	0.208	0.0012	447.9	3.1	0.5529	2.00	-0.9966	1	2.3	**4.894**	**0.117**
	0.184	0.0021	256.6	1.8	0.6654	0.67	-0.9738				
	0.217	0.0004	990.4	5.8	0.2524	15.15	-0.9990				
	0.217	0.0005	866.5	7.5	0.3158	14.11	-0.9996				
	0.207	0.0003	1233.7	10.9	0.1090	80.00	-0.9960				
483-10	0.194	0.0003	1001.4	8.3	0.1409	45.05	-0.9999	1A	0.67	**5.764**	**0.141**
	0.207	0.0003	985.4	10.2	0.1467	52.93	-0.9999				
**6N-645**											
645-1	0.768	0.0017	935.9	2.5	0.3991	3.15	-0.9996	1A	0.22	**3.812**	**0.183**
	3.285	0.0064	1048.2	4.5	0.3505	7.13	-0.9992				
645-3	0.562	0.0055	297.5	1.1	0.6623	0.39	-0.9900	1A	0.24	**4.997**	**0.276**
	0.771	0.0081	277.9	0.3	0.6741	0.07	-0.9900				
645-4	0.972	0.0016	1071.5	1.3	0.2936	2.70	-0.9976	1	0.87	**4.276**	**0.114**
	0.900	0.0012	1178.8	1.2	0.2376	3.46	-0.9980				
	0.930	0.0013	1177.6	2.0	0.2392	5.54	-0.9951				
645-5	0.993	0.0014	1206.6	1.0	0.2958	2.08	-0.9989	1A	0.93	**3.809**	**0.121**
	1.021	0.0011	1413.2	3.3	0.1990	11.95	-0.9862				
645-6	1.710	0.0047	794.7	1.1	0.4501	1.10	-0.9947	1A	0.83	**4.18**	**0.112**
	1.613	0.0011	1527.6	1.3	0.0745	14.82	-0.9930				
645-7	0.423	0.0057	218.3	0.6	0.6667	0.23	-0.9577	2A	226	**5.901**	**0.44**
	0.388	0.0009	730.9	1.8	0.2989	3.59	-0.9993				

	0.451	0.0011	753.8	1.6	0.3103	3.07	-0.9950				
645-8	0.311	0.0009	695.8	3.9	0.3576	6.05	-0.9990	1	0.15	**5.343**	**0.162**
	0.226	0.0004	919.4	9.5	0.2302	28.04	-0.9997				
	0.319	0.0017	469.9	2.8	0.4900	2.41	-0.9938				
645-9	0.413	0.0007	1101.8	7.1	0.2333	20.67	-0.9999	1A	0.29	**4.586**	**0.122**
	0.286	0.0010	709.4	5.2	0.4509	5.36	-0.9852				
645-10	0.513	0.0004	1456.9	7.1	0.0322	194.42	-0.9997	1A	0.4	**4.621**	**0.112**
	0.526	0.0004	1437.5	5.2	0.0401	113.15	-0.9998				
645-11	0.502	0.0005	1435.7	3.3	0.1461	17.02	-0.9997	1A	1.14		
	0.896	0.0008	1479.8	3.4	0.1240	21.61	-0.9986				
	0.843	0.0008	1448.6	6.2	0.1351	35.27	-0.9998				
645-12	0.279	0.0008	772.4	6.3	0.4298	7.02	-0.9995	1A	1.02	**4.525**	**0.127**
	0.262	0.0003	1501.0	23.5	0.0224	930.63	-1.0000				
645-13L	0.646	0.0013	854.0	1.5	0.2740	3.54	-0.9984	1	0.61	**5.243**	**0.117**
	0.615	0.0007	1142.5	3.1	0.1091	22.63	-0.9990				
	0.612	0.0020	638.5	0.9	0.3951	1.14	-0.9665				
645-13D	1.378	0.0011	1455.8	1.5	0.1137	10.77	-0.9962	1	1.8	**4.137**	**0.112**
	1.413	0.0009	1561.7	1.3	0.0655	16.03	-0.9905				
	1.444	0.0013	1399.9	1.3	0.1425	7.05	-0.9889				
	1.444	0.0011	1528.5	3.1	0.0805	31.95	-0.9963				
645-14	0.675	0.0005	1636.9	1.7	0.1445	8.86	-0.9984	1A	0.8	**3.567**	**0.114**
	0.689	0.0005	1700.5	2.0	0.1165	13.58	-0.9988				
645-15A	0.466	0.0060	239.1	0.9	0.7109	0.27	-0.9516	2	5.6	**3.61**	**0.181**
	0.446	0.0020	592.6	2.2	0.5610	1.34	-0.9965				
	0.452	0.0018	641.3	1.6	0.5461	1.10	-0.9965				
	0.459	0.0036	369.0	1.4	0.6566	0.54	-0.9880				
	0.445	0.0033	387.0	1.8	0.6477	0.71	-0.9932				
645-15B	0.596	0.0006	1574.6	5.0	0.1787	20.23	-0.9993	1	1.6	**3.474**	**0.133**
	0.608	0.0006	1560.1	4.1	0.1792	16.76	-0.9993				
	0.644	0.0006	1625.0	5.5	0.1486	28.02	-0.9996				
	0.579	0.0007	1440.0	4.7	0.2272	14.19	-0.9994				
	0.361	0.0005	1329.5	6.2	0.2673	14.80	-0.9997				
645-17	0.288	0.0002	1257.7	4.4	0.0355	108.53	-0.9990	1A	0.28	**5.302**	**0.112**
	0.242	0.0004	1003.3	6.9	0.1992	24.73	-0.9995				
645-18	1.550	0.0057	654.2	0.7	0.5098	0.56	-0.9603	1A	0.031	**4.042**	**0.114**
	1.155	0.0009	1509.2	1.9	0.1076	13.87	-0.9671				
645-19	1.358	0.0060	574.1	1.2	0.5379	0.83	-0.9941	1A	0.47	**4.005**	**0.151**
	1.070	0.0061	469.9	1.0	0.5853	0.57	-0.9900				
645-20	1.013	0.0033	718.9	2.1	0.4796	1.84	-0.9989	1A	0.63	**4.261**	**0.118**
	0.998	0.0048	536.5	0.9	0.5744	0.50	-0.9900				
645-21	0.989	0.0046	558.4	0.8	0.5823	0.45	-0.9560	1A	0.84	**4.028**	**0.112**
	1.006	0.0011	1319.3	1.7	0.2071	5.90	-0.9986				
**6N-700**											
LH-7a	0.737	0.0009	1132.8	1.5	0.1944	5.66	-0.9975	1	0.99	**4.849**	**0.112**
	0.732	0.0023	721.8	0.8	0.4612	0.83	-0.9756				
	0.756	0.0011	1077.4	1.7	0.2298	5.00	-0.9981				
**6N-1411**											
1411-2	0.469	0.0029	419.8	2.2	0.5341	1.58	-0.9991	1A	1.5	**5.241**	**0.124**
	0.599	0.0011	902.1	3.5	0.2530	8.96	-0.9976				
1411-3	0.929	0.0008	1749.4	1.0	0.2498	2.69	-0.9957	2A	108	**2.848**	**0.169**
	1.097	0.0022	1135.2	1.9	0.4622	1.83	-0.9994				
	1.095	0.0010	1740.9	1.8	0.2400	4.95	-0.9949				
1411-4	0.628	0.0003	2504.9	8.0	0.1041	61.73	-0.9993	2	7	**2.495**	**0.17**
	0.598	0.0025	690.3	2.8	0.6605	1.06	-0.9974				

	0.606	0.0004	2360.0	8.3	0.1569	39.60	-0.9973				
1411-6	0.337	0.0009	936.9	4.0	0.4824	3.52	-0.9987	1	1.11	**3.196**	**0.115**
	0.363	0.0008	1043.1	5.9	0.4442	6.19	-0.9996				
	0.341	0.0009	937.5	2.4	0.4819	2.17	-0.9988				
	0.355	0.0022	473.6	2.7	0.6587	1.01	-0.9995				
	0.376	0.0011	903.9	5.2	0.4979	4.32	-0.9996				
1411-9	2.148	0.0047	1115.2	0.7	0.5181	0.55	-0.9984	1A	0.41	**2.472**	**0.208**
	1.628	0.0010	2279.5	8.0	0.1693	2.65	0.9970				
	0.642	0.0003	2777.6	7.9	0.0336	204.85	-0.9999				
1411-10	1.508	0.0038	767.9	1.4	0.3570	1.53	-0.6980	1A	0.018	**5.128**	**0.122**
	0.152	0.0189	26.9	0.5	0.8029	0.12	-0.7011				
1411-11	0.450	0.0009	1081.3	1.5	0.4306	1.70	-0.9989	1A	1.05	**3.259**	**0.126**
	0.475	0.0012	965.5	3.2	0.4768	2.88	-0.9999				
**6N-1536**											
1536-1	0.761	0.0009	1259.5	2.7	0.1985	9.77	-0.9993	1A	0.092	**4.212**	**0.113**
	1.000	0.0006	1605.9	3.6	0.0297	106.01	-0.9983				
1536-2	1.412	0.0016	1348.1	1.1	0.2226	3.35	-0.9957				
	1.314	0.0012	1479.2	1.4	0.1678	6.27	-0.9970	1	1.9	**3.767**	**0.114**
	1.415	0.0018	1260.2	0.9	0.2612	2.17	-0.9958				
1536-3	7.149	0.0036	2686.6	1.8	0.1634	8.38	-0.9986	1A	0.85	**2.134**	**0.112**
	5.169	0.0078	1471.6	0.6	0.4468	0.62	-0.9979				
1536-4	2.975	0.0032	1567.2	1.0	0.2785	2.26	-0.9986	2A	9.5	**2.964**	**0.307**
	2.820	0.0026	1713.6	1.3	0.2392	3.62	-0.9979				
1536-5	2.836	0.0041	1212.7	0.9	0.3026	1.87	-0.9815	2	10.5	**3.762**	**0.126**
	2.540	0.0024	1476.3	1.7	0.1791	6.80	-0.9972				
	2.690	0.0029	1398.7	1.7	0.2169	5.43	-0.9965				
	2.754	0.0030	1380.6	1.1	0.2263	3.26	-0.9975				
1536-8	2.356	0.0015	1751.2	1.7	0.0966	13.89	-0.9993	2A	12	**3.502**	**0.395**
	2.451	0.0024	1503.3	1.5	0.2181	4.56	-0.9799				
1536-9	0.909	0.0007	1392.6	2.9	0.0914	26.34	-0.9994	2A	14	**4.445**	**0.117**
	1.340	0.0017	1194.5	3.0	0.2076	10.07	-0.9995				
	0.887	0.0007	1456.3	4.6	0.0582	67.75	-0.9998				
1536-10	0.509	0.0004	1444.4	5.4	0.0808	55.11	-0.9996	1A	1.4	**4.384**	**0.113**
	0.500	0.0009	1074.9	5.2	0.2823	11.51	-0.9997				
1536-11	2.901	0.0022	1845.7	1.5	0.1941	5.69	-0.9959	2A	17	**2.911**	**0.276**
	2.684	0.0024	1746.0	1.3	0.2393	3.62	-0.9184				
1536-13	1.485	0.0009	1599.4	1.5	0.0456	28.99	-0.9979	1A	0.0015	**4.144**	**0.112**
	1.431	0.0009	1579.7	2.3	0.0552	35.39	-0.9991				
	0.935	0.0006	1579.8	2.3	0.0502	38.54	-0.9964				
	1.086	0.0008	1520.5	3.1	0.0838	30.56	-0.9988				
1536-14	2.566	0.0019	1704.3	2.1	0.1375	12.05	-0.9953	2A	18	**3.458**	**0.114**
	2.693	0.0016	1821.8	1.9	0.0909	17.02	-0.9984				
	1.959	0.0010	1924.7	1.9	0.0420	39.70	-0.9987				
1536-18	0.139	0.0002	1250.4	14.2	0.2778	32.34	-0.9998	2	16	**3.735**	**0.478**
	0.128	0.0004	769.1	4.2	0.4750	3.85	-0.9965				
	0.124	0.0002	1353.8	9.2	0.2310	26.93	-0.9999				
	0.126	0.0004	806.9	5.4	0.4884	4.86	-0.9339				
**6N-1728**											
1728-1	1.323	0.0017	973.2	0.5	0.1454	2.58	-0.9570	2A	13	**5.832**	**0.475**
	1.365	0.0015	1056.8	2.2	0.1033	16.96	-0.9409				
1728-4	0.635	0.0015	725.2	1.3	0.3029	2.67	-0.9973	2	185	**6.274**	**0.267**
	0.568	0.0039	380.3	1.2	0.5831	0.65	-0.9900				
	0.705	0.0025	606.4	4.0	0.4061	4.98	-0.9992				
	0.571	0.0029	468.5	0.8	0.5157	0.63	-0.9979				
1728 5	0.449	0.0012	706.2	1.5	0.3140	2.79	-0.9992	2A	11.5	**6.074**	**0.749**
	0.477	0.0014	665.6	3.1	0.3527	4.85	-0.9782				
1728-6	0.647	0.0005	2471.5	5.2	0.3718	7.58	-0.9999	2	3.6	**1.598**	**0.12**

	0.657	0.0007	2100.6	5.7	0.4397	6.14	-0.9998				
	2.344	0.0104	672.6	0.4	0.6904	0.19	-0.6852				
	2.937	0.0030	2123.2	1.2	0.4394	1.33	-0.9993				
**6N-2098**											
FS-02	0.727	0.0007	1109.3	2.4	0.0765	26.11	-0.9983	1A	0.62	**5.702**	**0.113**
	0.614	0.0025	553.2	0.6	0.4658	0.61	-0.9972				
FS-03	0.867	0.0057	402.4	0.5	0.5765	0.31	-0.9842	2	7,8	**5.501**	**0.344**
	0.767	0.0074	297.5	0.4	0.6457	0.20	-0.9527				
	0.773	0.0036	514.9	1.0	0.5004	0.78	-0.9985				
FS-04	0.329	0.0034	282.9	0.8	0.6563	0.31	-0.9667	1	0.59	**5.663**	**0.117**
	0.345	0.0022	414.4	1.4	0.5666	1.07	-0.6831				
	0.423	0.0134	104.3	0.3	0.7806	0.11	-0.6748				
2098-14-1	0.183	0.0006	688.4	11.5	0.3557	17.85	-0.9998	1A	0.001	**5.743**	**0.222**
	0.176	0.0004	942.7	21.3	0.1833	84.26	-0.9998				
**6N-2200**											
2200-1	0.896	0.0006	1622.4	1.0	0.1032	7.93	-0.9996	1	1.8	**3.811**	**0.112**
	0.859	0.0007	1553.8	1.4	0.1362	7.91	-0.9996				
	0.908	0.0081	337.5	0.3	0.7143	0.08	-0.9990				
	1.333	0.0011	1519.1	0.9	0.1520	4.30	-0.9992				
	1.213	0.0008	1668.6	1.9	0.0808	19.00	-0.9998				
2200--2	1.251	0.0037	779.4	1.7	0.4731	1.73	-0.7433	2	6	**4.197**	**0.135**
	1.189	0.0012	1372.3	4.0	0.1585	19.04	-0.9947				
	1.180	0.0017	1183.4	3.2	0.2533	8.20	-0.9992				
	1.294	0.0020	1130.9	2.2	0.2826	4.93	-0.9674				
2200-4	3.387	0.0018	1984.1	1.0	0.0763	10.72	-0.9928	1A	0.17	**3.236**	**0.112**
	3.538	0.0022	1852.0	0.8	0.1272	4.82	-0.9785				
2200-6	3.180	0.0022	1748.4	1.4	0.1234	9.14	-0.9961	1A	1.06	**3.451**	**0.112**
	3.231	0.0034	1488.9	1.5	0.2340	4.23	-0.9971				
	3.032	0.0026	1610.2	1.4	0.1798	4.36	-0.7837				
2200-7	4.918	0.0028	1808.0	0.7	0.0815	6.60	-0.9811	2A	258	**3.445**	**0.179**
	4.489	0.0038	1591.6	1.1	0.1777	4.65	-0.9952				
	5.083	0.0040	1689.1	1.0	0.1697	4.34	-0.9145				
2200-8	0.815	0.0005	1680.9	3.1	0.0587	45.07	-0.9968	1	0.14	**3.871**	**0.116**
	0.742	0.0006	1550.5	4.2	0.1077	31.03	-0.9996				
	0.969	0.0006	1727.6	4.2	0.0437	83.26	-0.9997				
	0.846	0.0007	1550.4	4.3	0.1099	31.24	-0.9998				
2200-9	0.929	0.0007	1636.1	3.6	0.0909	32.77	-0.9993	1	1.3	**3.843**	**0.112**
	0.971	0.0007	1637.8	2.8	0.0889	26.02	-0.9991				
	0.981	0.0006	1709.7	3.7	0.0551	57.49	-0.9991				
	1.036	0.0013	1316.4	2.8	0.2467	7.47	-0.9764				
2200-11	1.131	0.0003	4809.1	4.5	0.1293	26.87	-0.9975	1A	0.14	**1.309**	**0.108**
	0.963	0.0003	4593.4	5.1	0.1623	23.63	-0.9803				
2200-CL1	6.196	0.0027	5285.9	4.0	0.4594	1.00	-0.9900	1A	0.26	**0.7**	**0.09**
	4.481	0.0008	8992.2	7.6	0.1897	28.71	-0.9995				
2200-CL2	4.432	0.0005	10333.4	3.4	0.1188	22.38	-0.9996	2	5.3	**0.681**	**0.089**
	6.145	0.0021	6045.3	1.3	0.4056	1.65	-0.9989				
	4.356	0.0097	1379.7	0.5	0.7189	0.13	-0.5948				
	6.863	0.0008	10753.5	5.0	0.0804	51.79	-0.9992				
2200-12-2	0.280	0.0027	337.5	1.3	0.7674	0.27	-0.9488	1A	0.033	**1.652**	**0.736**
	0.273	0.0012	703.3	3.6	0.6988	1.09	-0.9866				
2200-12-3	1.693	0.0011	1721.4	2.3	0.1067	17.59	-0.9977	1	2.6	**3.548**	**0.113**
	1.930	0.0010	1842.4	1.4	0.0579	20.22	-0.9956				
	1.383	0.0012	1544.9	1.2	0.1696	5.09	-0.9935				
2200-12-4	1.264	0.0040	732.5	1.5	0.4667	1.42	-0.9994	2	12	**4.164**	**0.124**
	1.161	0.0039	699.8	1.5	0.4793	1.34	-0.9990				
	1.107	0.0025	898.3	2.0	0.3873	2.75	-0.9996				
	1.185	0.0041	692.4	2.2	0.4833	1.95	-0.9997				
	1.236	0.0078	441.4	1.7	0.6114	0.81	-0.9977				

	1.159	0.0428	88.6	0.2	0.7818	0.10	-0.6037				
2200-13	0.228	0.0002	2982.5	10.2	0.4312	11.25	-0.9993	1	1.13	**1.174**	**0.113**
	0.305	0.0004	2204.1	9.7	0.5199	7.25	-0.9998				
	0.243	0.0003	2290.6	23.9	0.4967	19.82	-1.0000				
	0.333	0.0009	1175.4	5.2	0.6526	2.03	-0.9972				
2200-14b	4.125	0.0022	1829.3	0.8	0.0661	4.15	-0.3901	2A	48	**3.509**	**0.345**
	3.878	0.0022	1843.7	0.8	0.0811	7.94	-0.9873				
2200-17B	0.715	0.0007	1465.2	5.3	0.1671	23.33	-0.9984	1	1.18	**3.827**	**0.12**
	0.615	0.0010	1158.0	2.9	0.3026	5.72	-0.9992				
	0.645	0.0008	1355.7	5.0	0.2123	16.45	-0.9996				
2200-18	0.804	0.0032	774.2	3.5	0.7029	2.13	-0.1382	1A	0.0019	**1.391**	**0.365**
	0.669	0.0049	441.9	1.9	0.7542	0.40	-0.9790				
LBSC-02	3.667	0.0133	891.9	0.8	0.7615	0.18	-0.8805	2	26	**0.659**	**0.105**
	3.665	0.0151	780.2	0.7	0.7629	0.20	-0.5899				
	3.918	0.0143	885.6	0.4	0.7594	0.11	-0.8016				
	4.877	0.0051	2750.0	1.5	0.6369	0.65	-0.9902				
LBCT-01	2.190	0.0012	1907.0	1.8	0.0785	18.94	-0.9986	1	0.32	**3.358**	**0.112**
	2.185	0.0012	1892.3	1.4	0.0837	13.57	-0.9977				
	2.859	0.0058	1043.2	0.5	0.4306	0.54	-0.9963				
	3.095	0.0024	1701.5	0.7	0.1621	3.22	-0.9922				
	3.053	0.0030	1538.9	0.7	0.2284	2.03	-0.9922				
	2.792	0.0084	809.9	1.7	0.5289	2.00	-0.1000				
**6N-2121**											
LTC	0.497	0.0002	9832.9	28.8	0.4676	27.10	-0.9999	2	4.1	**0.412**	**0.066**
	0.487	0.0034	486.3	1.6	0.8094	0.33	-0.4949				
	0.511	0.0022	785.8	1.8	0.7965	0.28	-0.8455				
	0.499	0.0032	526.8	2.9	0.8133	0.63	-0.4708				
**6N-2233**											
2233-1	1.837	0.0017	1295.4	1.1	0.1374	6.84	-0.6874	2A	6.8	**4.535**	**0.231**
	1.335	0.0011	1346.2	1.8	0.1001	14.72	-0.9974				
**6N-2455**											
2455-1	1.195	0.0020	1007.4	1.6	0.2874	3.34	-0.9951	1A	0.27	**4.562**	**0.156**
	1.055	0.0024	874.5	2.3	0.3613	3.67	-0.8721				
2455-2	1.586	0.0022	1000.9	2.0	0.1680	7.50	-0.8582	1	2.5	**5.625**	**0.113**
	1.152	0.0018	945.9	2.0	0.2159	6.57	-0.9827				
	1.461	0.0016	1075.5	0.7	0.1175	4.94	-0.9908				
**6N-2953**											
2953-1	0.545	0.0028	454.5	2.5	0.4793	1.80	-0.7090	1	0.021	**6.419**	**0.118**
	0.672	0.0015	747.4	3.2	0.2548	8.41	-0.8588				
	0.569	0.0009	876.5	5.1	0.1571	24.32	-0.9996				
**6N-2996**											
	1.240	0.0020	1069.1	3.8	0.2683	8.77	-0.9643				
2296-1	1.576	0.0027	1032.4	1.9	0.2992	3.90	-0.9971	2A	14	**4.371**	**0.15**
	1.487	0.0054	648.8	1.5	0.4922	1.26	-0.9722				
	1.551	0.0072	550.6	1.1	0.5486	0.74	-0.9967				
2996-1core	0.899	0.0023	781.4	2.5	0.3726	4.39	-0.2340	1A	0.0062	**4.91**	**0.113**
	0.917	0.0008	1280.6	2.1	0.0821	21.48	-0.9857				
2996-2	0.162	0.0004	754.8	5.1	0.2449	13.82	-0.9996	1A	0.025	**6.46**	**0.237**
	0.129	0.0005	666.3	6.7	0.3112	12.13	-0.8665				
2996-3	0.501	0.0009	819.5	2.3	0.2237	7.09	-0.9988	1A	0.78	**6.152**	**0.209**
	0.261	0.0005	809.7	5.3	0.2283	15.85	-0.9995				
**5N-5203**	1.000	0.0012	1270.9	4.5	0.1825	17.68	-0.9935	1	0.03	**4.275**	**0.116**
	0.952	0.0017	1054.7	6.5	0.2897	13.89	-0.9970				
	0.904	0.0056	446.0	2.7	0.5891	1.41	-0.9924				
**5N-5334**											
5334-6b	0.385	0.0007	930.4	4.5	0.2772	10.29	-0.9976	1A	1.70	**5.098**	**0.115**
	0.398	0.0004	1290.7	8.5	0.0466	156.40	-0.9981				

**5N-5337**											
5337-4	0.606	0.0011	1093.1	2.7	0.3404	4.58	-0.9909	1	1.50	**3.909**	**0.115**
	0.564	0.0007	1327.9	5.3	0.2258	15.97	-0.9997				
	0.550	0.0012	991.6	4.7	0.3791	7.11	-0.7663				
**5N-5354**											
5354-2.1	0.513	0.0006	1361.8	5.8	0.1758	24.27	-0.9996				
5354-2.2	0.580	0.0015	896.2	3.8	0.4564	3.77	-0.9548				
5354-2.3	1.089	0.0010	1532.3	3.4	0.1693	15.05	-0.9851	1	0.02	**3.700**	**0.113**
	0.863	0.0006	1711.0	3.1	0.0858	29.60	-0.9856				
	1.115	0.0007	1732.6	3.3	0.0761	36.15	-0.9882				
**5N-5708**											
5708-1	0.830	0.0008	1175.9	4.3	0.0563	65.14	-0.9929	2A	7.30	**5.488**	**0.214**
	0.849	0.0009	1151.3	2.6	0.0821	25.86	-0.9778				
5708-3	0.337	0.0115	94.5	1.0	0.7574	0.19	-0.7721	1A	0.05	**6.566**	**0.366**
	0.343	0.0168	66.7	0.6	0.7792	0.12	-0.4219				
**5N-5849**	0.367	0.0010	741.5	6.1	0.3250	10.84	-0.9960	1A	0.44	**5.59**	**0.162**
	0.399	0.0006	1032.7	12.3	0.1401	67.62	-0.9990				
**5N-5887**	1.083	0.0076	376.1	0.9	0.5840	0.45	-0.8300	1A	0.87	**5.886**	**0.22**
	1.009	0.0059	440.2	1.1	0.5372	0.65	-0.8583				
**5N-6801**											
	2.156	0.0019	2146.7	2.6	0.3186	4.74	-0.9796	1A	0.36	**2.047**	**0.15**
	2.043	0.0017	2187.0	2.1	0.3104	4.01	-0.9900				

Supplementary Table 2. U-Pb isotopic and age data for straw stalactites and helictites from cave 6N-370.
Ages are corrected for initial disequilibrium in the decay chain using 234/238i = 1±0.3.

Sample ID	U ppm	Pb ppm	$^{238}U^{206}Pb$	% err	$^{207}Pb^{206}Pb$	% err	Corr. coeff.	Isochron type	MSWD	Age (Ma)	uncertainty 2σ
370-S1	0.564	0.0024	560.2	1.3	0.4828	1.02	-0.9173	2	201	**5.08**	**0.6**
	0.516	0.0010	861.7	1.7	0.2890	3.53	-0.9562				
	0.477	0.0019	560.5	1.9	0.4556	1.82	-0.9631				
	0.440	0.0025	430.4	1.1	0.5094	0.78	-0.8907				
	0.478	0.0012	750.5	1.9	0.3428	3.03	-0.9515				
	0.532	0.0012	807.5	2.2	0.3298	3.79	-0.9729				
	0.737	0.0029	581.2	1.6	0.4655	1.41	-0.9464				
370-S2	0.451	0.0012	750.2	1.6	0.3514	2.38	-0.9479	2	3.3	**5.3**	**0.13**
	0.498	0.0007	1031.9	2.4	0.1786	9.51	-0.9768				
	0.476	0.0012	772.6	1.3	0.3410	1.97	-0.9202				
	0.469	0.0006	1070.1	2.6	0.1485	12.88	-0.9791				
	0.526	0.0009	946.9	2.3	0.2320	6.56	-0.9755				
	0.481	0.0007	1026.9	3.3	0.1845	12.67	-0.9870				
	0.467	0.0007	1003.9	3.2	0.1992	11.35	-0.9875				
370-S3	0.200	0.0014	388.4	2.6	0.5643	1.55	-0.9803	2	19	**5.34**	**0.14**
	0.279	0.0004	1013.9	4.7	0.1831	18.68	-0.9942				
	0.287	0.0006	877.4	2.3	0.2776	5.11	-0.9750				
	0.211	0.0003	1015.1	4.3	0.1787	17.36	-0.9929				
	0.189	0.0015	349.6	1.3	0.5972	0.63	-0.9900				
	0.200	0.0007	659.8	4.7	0.3877	6.33	-0.9912				
	0.310	0.0005	1011.8	6.3	0.1779	25.69	-0.9967				
370-S4	0.329	0.0015	502.8	1.3	0.4726	1.14	-0.9242	2	9.9	**5.24**	**0.15**
	0.316	0.0014	525.8	2.1	0.4459	2.16	-0.9716				
	0.328	0.0005	1032.6	3.5	0.1705	14.91	-0.9892				
	0.233	0.0006	750.9	6.7	0.3166	12.44	-0.9970				
	0.325	0.0011	616.6	2.0	0.4077	2.36	-0.9675				
	0.271	0.0005	885.6	5.5	0.2634	13.53	-0.9958				
	0.319	0.0003	1214.9	4.9	0.0681	60.17	-0.9943				
370-S5	0.331	0.0004	1093.2	4.3	0.1374	24.20	-0.9929	2	2.4	**5.3**	**0.12**
	0.418	0.0023	450.6	1.5	0.5299	1.02	-0.9412				
	0.378	0.0006	981.4	2.0	0.2087	6.57	-0.9676				
	0.410	0.0013	671.5	2.0	0.3975	2.52	-0.9683				
	0.304	0.0006	899.9	5.8	0.2517	14.99	-0.9961				
	0.413	0.0014	645.3	1.6	0.4151	1.82	-0.9492				
	0.384	0.0018	509.0	1.8	0.4933	1.46	-0.9579				

Supplementary Table 3. U-Th data for Nullarbor materials with measurable ^{234}U disequilibrium

Sample	Lab number	$[^{230}Th/^{238}U]^a$	$[^{234}U/^{238}U]^a$	$[^{232}Th/^{238}U]$	$[^{230}Th/^{232}Th]$	Age(ka)^b	$[^{234}U/^{238}U]_i^c$
LBC Cor 03b.1	UMA00824 Nov-2004	0.8887(34)	0.9366(32)	0.03570(13)	24.9	388(30)	0.809(25)
LTC1.1	UMA00825 Nov-2004	0.7188(22)	0.8812(23)	0.007799(77)	92.2	200(3)	0.7904(55)
LTC stal ca. 90mm	UMA00826 Nov-2004	0.8738(36)	0.9314(28)	0.002974(14)	294	360(19)	0.809(16)
LBCSC01.1	UMA00848 Nov-2004	0.9063(44)	1.0016(25)	0.03040(23)	29.8	250.8(7.9)	1.0029(50)
LBCSC01.2	UMA00865 Nov-2004	1.0172(34)	1.0304(23)	0.0005346(32)	1903	410(20)	1.0972(55)
CL-1	UMB03830 Jan-2011	1.0256(38)	1.0224(22)	0.0003056(56)	3356	569(106)	1.113(38)
CL-2	UMB03847 Jan-2011	1.0332(41)	1.0285(22)	0.0000258(14)	40079	554(91)	1.137(36)
2200-19	UMD120411-188	1.0048(28)	1.0089(37)	0.0010836(41)	927	540(103)	1.040(11)
84-05	UMD120411-219	0.9895(34)	0.9985(41)	0.003563(13)	278	531(115)	0.992(23)
2953-01	UMD120411-254	0.9085(25)	1.0038(40)	0.0021447(52)	424	255.3(5.9)	1.0078(81)
2200-sc-02	UMD120411-303	1.0196(26)	1.0221(39)	0.0004463(23)	2285	496(61)	1.0894(77)
2200-LTC	UMD120411-307	0.8561(26)	0.9211(37)	0.0003104(13)	2758	344(17)	0.792(19)
LTC 'tite tip	UMD130410-536	0.9250(41)	0.9758(26)	0.0011077(74)	835	340(14)	0.9367(86)

^{a} Activity ratios determined after Hellstrom (2003)

^{b} Age corrected for intial ^{230}Th using eqn. 1 of Hellstrom (2006), the decay constants of Cheng et al (2013) and $[^{230}Th/^{232}Th]_i$ of 1.5 ± 1.5

^{c} Initial $[^{234}U/^{238}U]$ calculated using corrected age

2-σ uncertainties in brackets are of the last two significant figures presented
